# Accuracy of stress perfusion cardiac magnetic resonance imaging in a district hospital

**DOI:** 10.1177/20584601231157018

**Published:** 2023-02-27

**Authors:** Jostein Gleditsch, Bjørn A Halvorsen, Konstantinos Bratis, Astrid D Alvim, Anders Jordal, Jan G Fjeld, Nezar Raouf, Sohail Aslam, Eike Nagel, Christian Hall

**Affiliations:** 1 60517 Østfold Hospital, Norway; 2 60517 University of Oslo, Norway; 3 60517 Østfold Hospital, Norway; 4 9173 Goethe University, Germany; 5 60517 Oslo University Hospital, Norway; 6 Brynklinikken Fastlegesenter, Norway; 7 Ringerike Hospital, Norway

**Keywords:** magnetic resonance imaging, cardiac imaging techniques, perfusion imaging, coronary disease, myocardial ischemia

## Abstract

**Background:**

The European Society of Cardiology has published updated guidelines regarding pathways for diagnosis and management of obstructive coronary artery disease (CAD). Non-invasive functional assessment, for example, by stress perfusion cardiac magnetic resonance (stress pCMR) is recommended in patients with intermediate pretest probability of disease. Previous pCMR studies were mainly performed in high volume university hospitals with experienced radiologists or cardiologists interpreting the images.

**Purpose:**

The aim of the present study was to evaluate the feasibility of establishing a stress pCMR imaging service in a district hospital.

**Material and Methods:**

One hundred and thirteen patients with intermediate pretest probability of CAD referred for single-photon emission computed tomography (SPECT) at the regional hospital also underwent adenosine stress pCMR locally. The diagnostic analysis was compared to that of an experienced cardiac magnetic resonance (CMR) center serving as a reference.

**Results:**

Inter-rater agreement between local readers and the reference reader was substantial to perfect for late gadolinium enhancement (LGE) (weighted kappa = 0.76 and 0.82), but only fair to moderate for pCMR (*k* = 0.34 and 0.51). No improvement in agreement between reference reader and local reader during the study was demonstrated.

**Conclusion:**

CMR is feasible in patients with intermediate pretest probability of obstructive CAD in the setting of a district hospital. However, as opposed to infarct detection with LGE, the interpretation of stress pCMR was more challenging. To establish this method, we suggest obtaining experience in close collaboration with a reference CMR center.

## Introduction

The European Society of Cardiology (ESC) recently updated their guidelines for the diagnosis and management of obstructive coronary artery disease (CAD).^1^ For patients with a high pretest probability (PTP) of CAD, direct referral to invasive coronary angiography (ICA), possibly with revascularization in the same session, is recommended. For patients in the lower range of PTP, a non-invasive diagnostic approach is advised. When a low clinical likelihood of CAD is suspected and patient characteristics suggest high image quality, anatomical assessment by computed tomography angiography of the coronary arteries (cCTA) is the preferred diagnostic pathway. In cases of intermediate or higher likelihood of disease and when revascularization is contemplated, a functional imaging test is recommended either by single-photon emission computed tomography (SPECT) or by stress perfusion cardiac magnetic resonance (stress pCMR).

Studies have documented that pCMR correlates better with findings of ICA than SPECT.^2,3^ Furthermore, in contrast to SPECT, stress pCMR has the advantage of no ionizing radiation,^4^ no attenuation artifacts, and better spatial resolution. Previous studies comparing SPECT and stress pCMR were mainly performed in high volume university hospitals with experienced radiologists or cardiologists interpreting the images.

Currently, pCMR for diagnostic work-up in suspected CAD is not in routine use in Norway. This may be due to diagnostic traditions, lack of necessary equipment, MRI capacity, local expertise, or expected need for extra resources when compared to SPECT, which is the more widespread method.

Successful implementation of a cCTA service in a district hospital setting has been demonstrated.^5^ In the present study, we evaluated the feasibility of establishing a service of stress pCMR. We compared our in-house interpretation of obtained images to that of an experienced CMR center.

## Methods

### Design and study population

This single center cohort study was conducted at a Norwegian district hospital serving 300.000 inhabitants. All participants provided written informed consent prior to entering the study. The study was approved by the Regional Committee for Medical and Health Research Ethics in Norway (REK 2010/3219).

Patients were recruited from the outpatient cardiology clinic in our hospital and from four practicing cardiologists in our catchment area in the period of April 2011 to August 2013. The criterion for inclusion into the study was a planned referral for SPECT. Indications for SPECT were either evaluation of suspected symptomatic stable CAD in patients with intermediate pretest probability of obstructive CAD or evaluation of a previously demonstrated borderline coronary artery stenosis. Pretest probability of obstructive CAD was evaluated at the discretion of the referring clinician.

Exclusion criteria were atrial fibrillation, previous coronary artery bypass surgery, severe renal failure (estimated glomerular filtration rate <30 mL/min/1.73 m^2^), communication problems, claustrophobia, inability to lie in a supine position for an hour, age below 30 or above 75, or contraindications to CMR or adenosine.

After inclusion, the patients underwent CMR. We assessed infarct by late gadolinium enhancement (LGE) and ischemia by adenosine stress pCMR. Subsequently, the patients were examined with SPECT. We did not record changes in symptoms or medication in the period between the two examinations.

Between March 2019 and April 2020, all CMR examinations were retrospectively reassessed by two local radiologists and one cardiologist at the CMR reference center (Division of Cardiovascular Imaging, Goethe University, Frankfurt am Main, Germany). After 10, 20, and 50 cases, respectively, we held telephone conferences between local and reference readers to facilitate learning. We did not retrospectively alter diagnostic evaluations after these sessions. The readers were blinded to patient history and to the other reader’s conclusions. The local radiologists had assessed 318 and 399 non-perfusion CMR respectively before start of this project, and had been consultants for 10 and 11 years. However, they had no prior experience in the performance and interpretation of pCMR. The reference reader had 10 years of CMR experience, reporting more than 400 cases annually.

Pretest probability of CAD according to the ESC 2019 guidelines^1^ was calculated retrospectively based on registered symptoms at time of inclusion.

### Cardiac magnetic resonance examination

The CMR examinations were performed with a 1.5 Tesla Siemens Avanto scanner (Siemens AG, Erlangen, Germany). For dynamic stress, all patients received an adenosine infusion of 140 μg/kg body weight/minute. After 3 minutes of infusion, a contrast injection with 0.05 mmol/kg dimeglumine gadopentetate (Dotarem – Guerbet, Paris, France) at a flow rate of 5 mL/s was started. Simultaneously, the stress pCMR image acquisition was initiated. The patient was asked to stop breathing when contrast arrived in right ventricle. The image acquisition lasted for 120 heartbeats with three short-axis slices sampled on each heartbeat. Adenosine infusion was stopped after approx. 50 heartbeats. We applied the following imaging parameters: Saturation recovery segmented gradient echo pulse sequence with TR/TE/TI of 167/1.11/120 ms, 108 × 144 matrix, 340–430 mm field of view, 1 NEX, 8 mm slice thickness.

After 10 minutes, rest of the pCMR imaging was performed without adenosine infusion, but with otherwise identical contrast parameters and pulse sequence parameters.

After another 10 minutes, LGE imaging was performed with 3 long-axis slices and short-axis slices that covered the entire left ventricle. Imaging parameters are phase sensitive gradient echo pulse sequence triggered on every second heart beat in diastolic phase with typical TR/TE/TI of 800/3.33/300 ms, 156 × 256 matrix, 330 mm field of view, 1 NEX, 8 mm slice thickness.

For CMR, a 16 segment score sheet, where the apical part had been excluded from a 17 segment sheet,^6^ was filled in for all patients. Each segment was assessed for perfusion and late gadolinium enhancement (LGE) pathology. The diagnosis of perfusion defect was based on a visual analysis with comparison between regions to identify relative hypo-perfusion according to current guidelines from the Society of Cardiovascular Magnetic Resonance.^7^ A comparison between stress and rest images was performed to identify inducible perfusion defects and artifacts. Perfusion defects in regions with LGE were only interpreted as reversible if the extent of the perfusion defect was clearly beyond the extent of LGE.

For evaluation of ischemia and infarct, a score was assigned to each segment indicating a normal (0), borderline (1), or pathologic (2) finding. The final overall score for LGE was deemed pathologic if at least one of the segments was classified as pathologic with a high degree of confidence. Similarly, the final score for pCMR was deemed pathologic if there was at least one segment with a high confidence of transmural pathology or two adjacent segments with high confidence of subendocardial pathology.^8^

### Single-photon emission computed tomography examination

Gated SPECT was performed with a dual head cardiac gamma camera (Ventri H3000YT, GE Healthcare, Chicago, Illinois, USA), with low energy high resolution collimators. Stress and rest studies were done in a one-day protocol using 99mTc-tetrofosmin (MYOVIEW^R^, GE Healthcare). For stress, bicycle exercise testing was performed in 96 patients. In 20 patients not able to perform bicycle exercise, adenosine stress was applied.

A 16 segment score sheet, where the apical part had been excluded from a 17 segment sheet,^6^ was applied. A fixed perfusion defect was interpreted as an infarct and a reversible perfusion defect as ischemia.

### Statistical analysis

Clinical characteristics were expressed as percentages or medians with inter quartile range as appropriate. Agreement between local and reference CMR center interpretations was calculated by using the weighted Cohen’s kappa (k). Confidence intervals for the kappa statistics were calculated from 2000 bootstrap replications. The level of agreement for a kappa value 0–0.20 was interpreted as slight, 0.21–0.40 fair, 0.41–0.60 moderate, 0.60–0.79 substantial, and 0.81–1.00 almost perfect.^9^ To identify possible improvements in agreement between reference reader and local readers during the study period, the patient cohort was stratified into two groups depending on when the images were analyzed, and compared with *z*-test. All statistics was calculated with Stata/SE v. 16.1 (StataCorp LLC, TX, USA).

## Results

A total of 113 patients were included in the study. Table 1 lists the demographic and clinical characteristics. Median age was 61 years (IQR: 51–68 years) and 64% were men. Thirty-seven percent had a history of previous CAD. Median pretest probability of CAD was 19% (IQR: 11–32%).

**Table 1.** Clinical characteristics, history of coronary artery disease, and symptoms of 113 patients examined with cardiac magnetic resonance, median (IQR), or number (proportion) as appropriate.

Number of patients	113
Men, *n* (%)	72 (64%)
Age, year, median (IQR)	61 (51–68)
Body Mass index, kg/m^2^, median (IQR)	27 (25–30)
Caucasian, *n* (%)	109 (94%)
Never smoked, *n* (%)	36 (32%)
Ex-smoker, *n* (%)	46 (41%)
Current smoker, *n* (%)	31 (27%)
Hypertension, *n* (%)	62 (55%)
Diabetes, *n* (%)	22 (19%)
History of coronary artery disease (CAD)	
Family history of premature CAD, *n* (%)	47 (42%)
Previous coronary artery disease, *n* (%)	42 (37%)
• Myocardial infarction, *n* (%)	21 (19%)
• Angina (not infarction), *n* (%)	21 (19%)
• Previous PCI, *n* (%)	31 (27%)
Symptoms at inclusion	
Typical angina, *n* (%)	46 (41%)
Atypical angina, *n* (%)	28 (25%)
Non-anginal chest pain, *n* (%)	13 (12%)
Asymptomatic, *n* (%)	11 (10%)
Dyspnea, *n* (%)	22 (19%)

### Cardiac magnetic resonance

Table 2 displays the LGE findings for local readers and reference reader in a cross-tab format. There was agreement with the reference examiner in 101/113 cases (reader 1) and in 106/113 cases (reader 2). Four cases were assessed as pathologic by reader 1 and negative by the reference reader. Only one case was deemed pathologic by reader 2, but not by the reference reader. Two and four cases were respectively judged as pathologic by the reference reader while diagnosed as normal by the local readers. The weighted kappa was 0.76 (95% CI: 0.61–0.90) for reader 1 and 0.82 (95% CI: 0.68–0.96) for reader 2.

**Table 2.** Late Gadolinium Enhancement findings. Cross-tab comparison of reference reader versus two local readers. Findings classified as negative, borderline or pathologic (defined as at least one segment with high confidence of pathologic finding). Cohen’s weighted kappa with 95% confidence intervals.

	Local reader 1			Local reader 2			Sum cases
Reference reader	Negative	Borderline	Pathologic	Negative	Borderline	Pathologic	
Negative	82	5	4	89	1	1	91
Borderline	1	0	0	1	0	0	1
Pathologic	2	0	19	4	0	17	21
Sum cases	85	5	23	94	1	8	113
Weighted kappa	0.76 (0.61–0.90)			0.82 (0.68–0.96)			

With regard to the perfusion findings (Table 3), there was agreement in 72 (reader 1) and 61 (reader 2) out of 113 cases. Two cases were assessed as pathologic by reader 1 and negative by the reference reader. Similarly, four cases judged by reader 2 as pathologic were evaluated as negative by the reference reader. Twelve and nineteen cases respectively were judged as pathologic by the reference reader while diagnosed as normal by the local readers. The weighted kappa was 0.50 (95% CI: 0.38–0.64) for reader 1 and 0.33 (95% CI: 0.20–0.48) for reader 2.

**Table 3.** Stress perfusion cardiac magnetic resonance findings. Cross-tab comparison of reference reader versus two local readers. Findings classified as negative, borderline or pathologic (defined as at least one segment with high confidence of transmural pathology or at least two adjacent segments with high confidence of subsegmental pathology). Cohen’s weighted kappa with 95% confidence intervals.

	Local reader 1			Local reader 2			Sum cases
Reference reader	Negative	Borderline	Pathologic	Negative	Borderline	Pathologic	
Negative	34	4	2	32	4	4	40
Borderline	9	4	7	10	1	9	20
Pathologic	12	7	34	19	6	28	53
Sum cases	55	15	43	61	11	41	113
Weighted kappa	0.51 (0.38–0.64)			0.34 (0.20–0.48)			

There was no statistically significant change in agreement either for LGE or stress pCMR during the study period when comparing Cohen’s kappa for the first 56 patients vs. the last 57 patients (Table 4).

**Table 4.** Agreement between reference reader versus two local readers stratified by first 56 patients and last 57 patients. Late Gadolinium Enhancement and stress perfusion cardiac magnetic resonance findings. Cohen’s weighted kappa with 95% confidence intervals. There were no significant differences between the first 56 vs. the last 57 patients (*z*-test).

	First 56 patients	Last 57 patients
LGE		
Local reader 1	0.69 (0.50–0.88)	0.78 (0.60–0.96)
Local reader 2	0.71 (0.55–0.89)	0.83 (0.66–0.99)
Stress pCMR		
Local reader 1	0.58 (0.40–0.77)	0.43 (0.25–0.61)
Local reader 2	0.44 (0.25–0.63)	0.24 (0.04–0.44)

Abbreviations: LGE: Late Gadolinium Enhancement, pCMR: perfusion cardiac magnetic resonance.

### Single-photon emission computed tomography

SPECT was performed in 107 of 113 patients. Median time between CMR and SEPCT was 33 days (IQR: 20–47 days). Two patients were not referred to SPECT for unknown reasons. Two patients did not attend to SPECT in spite of referral. Two patient developed symptoms of acute coronary syndrome while waiting for SPECT. Both of these patients were referred to ICA and had percutaneous transluminal coronary angioplasty (PTCA) performed. As shown in Table 5, 89/107 (84%) patients had no pathologic findings on SPECT.

**Table 5.** SPECT and angiography findings in 113 patients examined with Cardiac Magnetic resonance, median (IQR) or number (proportion). CT angiography or invasive coronary angiography within 1 year.

SPECT-findings (*n* = 107)	
Negative	89 (84%)
Scar/fixed defect	8 (7%)
Reversible defect	5 (5%)
Combination	5 (5%)
CT coronary angiography (n = 1)	
No significant stenosis detected	1
Stenosis detected	0
Invasive coronary angiography (n = 16)	
No significant stenosis detected	8 (50%)
Stenosis detected	8 (50%)

### Angiography

ICA was performed within 12 months in a total of 16/113 CMR patients and 14/107 SPECT patients (Table 5). Only one patient performed cCTA. Among those who performed ICA, 6/14 had a pathologic finding on SPECT, and 12/16 a pathologic finding on CMR according to reference reader. Eight patients out of these sixteen had significant coronary stenosis and underwent revascularization (3/6 had a pathologic finding on SPECT and 6/8 had pathologic finding on CMR).

## Discussion

In this study, we examined the feasibility of establishing a perfusion CMR imaging service in a district hospital. The locally obtained diagnostic results were compared to that of readings at an experienced CMR center serving as a reference. Although it was expected that the agreement would be better for conventional CMR (where the local readers had experience) than for stress perfusion pCMR, we considered it relevant to record the level of agreement between a reference center and a district hospital after a limited amount of training. We consider our results to be relevant to other district hospitals that consider starting a stress pCMR service as they might illustrate how guidelines may be implemented in practice.

The inter-rater agreement analysis showed substantial to perfect agreement for diagnosis of infarction with LGE (*k* = 0.76 and 0.82), but only fair to moderate agreement for diagnosis of ischemia with pCMR (*k* = 0.34 and 0.51). Furthermore, pCMR agreement was not improved during the study period. In contrast to the high accuracy and reliability of CMR in cardiac function and LGE imaging, pCMR is known to be adversely affected by multiple factors during data acquisition as well as post-processing.^10^ Chih et al.^11^ previously found a high interobserver reproducibility (coefficient of variation 9%, *r* = 0.93) of stress pCMR in patients with multi-vessel CAD and low risk for CAD. Muhling et al. found good interobserver agreement using semi-quantitative analysis provided the quality of images was good.^12^ However, in the MR-IMPACT study^13^, the reader agreement was only fair, with a kappa of 0.30–0.39 which is in line with our results. The present study expands the current literature regarding the interobserver agreement of pCMR imaging in a district hospital. Although not directly underpinned by our results, we speculate that the results would further improve by continued training. However, more cases than we had available in our study are necessary to ensure adequate training in stress pCMR.

We found a higher number of borderline cases in pCMR compared to LGE, both for the reference reader and for the two local readers. This can be explained by the complexity in the pCMR interpretation. As opposed to LGE, the subtle contrast difference between normal and pathologic myocardium in pCMR is more demanding to perceive and increases the probability of disagreement between readers. Furthermore, extensive perfusion defects in which all diagnostic criteria are met were not common in our study population. Collectively, these factors may have contributed to the lower interobserver agreement for pCMR compared to LGE.

At the time of inclusion in our study, ESC guidelines recommended testing for ischemia in symptomatic patients with an intermediate pretest probability of obstructive CAD. In this study, the clinicians assessed pretest probability at their own discretion, applying risk estimation methods as they saw fit. Post-hoc analysis using the ESC 2019 guidelines table showed a median pretest probability of CAD in our patient group of 19%, confirming inclusion of a clinically relevant population. The prevalence of ischemia was 53/113 (47%) by CMR (according to the reference reader). In contrast, only 10/107 (9%) had ischemia as diagnosed by SPECT. Collectively, these data may indicate that there might be a substantial proportion of false negative SPECT examinations in our study and/or the CMR readings were too sensitive.

In a district hospital setting, the prevalence of obstructive coronary artery disease is expected to be lower than in university hospitals receiving more selected patient groups. Standard imaging to rule out CAD in our region is cCTA or SPECT depending on clinical probability. We suspected a significant referral bias in our material. Direct referral to ICA bypassing other non-invasive imaging tests may have influenced our results. However, the prevalence of ischemia according to the reference reader was comparable with expected pretest probability of CAD. Furthermore, it is unlikely that a selection bias has affected our results when it comes to the comparison between local readers and reference readers.

One of the main strengths of pCMR is the detection of microvascular angina due to coronary microvascular dysfunction (MvD) in the setting of non-obstructive coronary artery disease. Although MvD would account for the patients’ symptomatology in up to 50% of all angina cases and comes with an increased risk of major adverse cardiovascular events,^14^ it has been a particularly vexing syndrome for clinicians to evaluate and manage, as there are no widely available non-invasive imaging tests that can identify such patients with reasonable accuracy.

Patients with MvD can safely be assessed using pCMR, both with quantification but also visually, in which case the entity presents as a global circumferential subendocardial defect at stress. As the accuracy of our local experience would only be optimally assessed against positron emission tomography (PET) perfusion quantification or more sophisticated invasive coronary measurements (e.g., index of microcirculatory resistance), it is not possible to establish the exact extent of MvD in our cohort. However, in 15/113 cases analyzed by the reference reader, a perfusion pattern suggestive of microvascular dysfunction was identified. This finding should be carefully taken into consideration in the interpretation of our results, as pCMR is superior to SPECT for the detection of MvD,^15^ and this would obviously not be confirmed with angiography, which is expected to be unremarkable in these cases. As such, the potentially increased detection of MvD with pCMR could be one of the major confounding parameters leading to significant discrepancy between the patient’s symptoms, pCMR, SPECT, and angiographic findings.

There are limitations in this study. This was a single center study involving only one reference reader and two local readers. However, the reference reader had completed training at a large-volume cardiovascular magnetic resonance core lab (Division of Cardiovascular Imaging, Goethe University, Frankfurt am Main, Germany). Nevertheless, involving multiple reference readers might have strengthened our results further by reducing the possibility of individual reader bias. Furthermore, all CMR examinations were performed in the period 2011–2013. Modern scanners have improved image quality. Improved image quality would probably have contributed to improved interobserver agreement, especially in pCMR.

In conclusion, we hold it feasible to establish and run a pCMR service at a district hospital. We conclude that the diagnosis of ischemia by stress pCMR was more challenging than LGE imaging. To secure sufficient quality in pCMR imaging and interpretation, we hold it essential that a close collaboration with a reference center is established to attain appropriate experience in image analysis.

## References

[1] Saraste A Knuuti J. ESC 2019 guidelines for the diagnosis and management of chronic coronary syndromes: recommendations for cardiovascular imaging. Herz 2020; 45: 409–420. 32430520 10.1007/s00059-020-04935-x PMC7391397

[2] Schwitter J Wacker CM Wilke N, et al. MR-IMPACT II: magnetic resonance imaging for myocardial perfusion assessment in coronary artery disease trial: perfusion-cardiac magnetic resonance vs. single-photon emission computed tomography for the detection of coronary artery disease: a comparative multicentre, multivendor trial. Eur Heart J 2013; 34: 775–781. 22390914 10.1093/eurheartj/ehs022

[3] Greenwood JP Maredia N Younger JF, et al. Cardiovascular magnetic resonance and single-photon emission computed tomography for diagnosis of coronary heart disease (CE-MARC): a prospective trial. Lancet 2012; 379: 453–460. 22196944 10.1016/S0140-6736(11)61335-4 PMC3273722

[4] Picano E Vano E Rehani MM, et al. The appropriate and justified use of medical radiation in cardiovascular imaging: a position document of the ESC associations of cardiovascular imaging, percutaneous cardiovascular interventions and electrophysiology. Eur Heart J 2014; 35: 665–672. 24401558 10.1093/eurheartj/eht394

[5] Halvorsen BA Rodevand O Hagen G, et al. Angiography with 64-channel CT upon suspicion of stable coronary disease. Tidsskr Nor Laegeforen 2008; 128: 2172–2176. 18846139

[6] Cerqueira MD Weissman NJ Dilsizian V, et al. Standardized myocardial segmentation and nomenclature for tomographic imaging of the heart. A statement for healthcare professionals from the cardiac imaging committee of the council on clinical cardiology of the American heart association. Int J Cardiovasc Imaging 2002; 18: 539–542. 12135124

[7] Schulz-Menger J Bluemke DA Bremerich J, et al. Standardized image interpretation and post-processing in cardiovascular magnetic resonance - 2020 update : Society for Cardiovascular Magnetic Resonance (SCMR): Board of Trustees Task Force on Standardized Post-Processing. J Cardiovasc Magn Reson 2020; 22: 19. 32160925 10.1186/s12968-020-00610-6 PMC7066763

[8] Nagel E Greenwood JP McCann GP, et al. Magnetic resonance perfusion or fractional flow reserve in coronary disease. N Engl J Med 2019; 380: 2418–2428. 31216398 10.1056/NEJMoa1716734

[9] Landis JR Koch GG. The measurement of observer agreement for categorical data. Biometrics 1977; 33: 159–174. 843571

[10] Bratis K Nagel E Variability in quantitative cardiac magnetic resonance perfusion analysis. J Thorac Dis 2013; 5: 357–359. 23825774 10.3978/j.issn.2072-1439.2013.06.08 PMC3698280

[11] Chih S Macdonald PS Feneley MP, et al. Reproducibility of adenosine stress cardiovascular magnetic resonance in multi-vessel symptomatic coronary artery disease. J Cardiovasc Magn Reson 2010; 12: 42. 20663155 10.1186/1532-429X-12-42 PMC2914773

[12] Muhling OM Dickson ME Zenovich A, et al. Quantitative magnetic resonance first-pass perfusion analysis: inter- and intraobserver agreement. J Cardiovasc Magn Reson 2001; 3: 247–256. 11816621 10.1081/jcmr-100107473

[13] Schwitter J Wacker CM van Rossum AC, et al. MR-IMPACT: comparison of perfusion-cardiac magnetic resonance with single-photon emission computed tomography for the detection of coronary artery disease in a multicentre, multivendor, randomized trial. Eur Heart J 2008; 29: 480–489. 18208849 10.1093/eurheartj/ehm617

[14] Hoffmann U Ferencik M Udelson JE, et al. Prognostic value of noninvasive cardiovascular testing in patients with stable chest pain: insights from the PROMISE trial (Prospective Multicenter Imaging Study for Evaluation of Chest Pain). Circulation 2017; 135: 2320–2332. 28389572 10.1161/CIRCULATIONAHA.116.024360 PMC5946057

[15] Mathew RC Bourque JM Salerno M, et al. Cardiovascular imaging techniques to assess microvascular dysfunction. JACC Cardiovasc Imaging 2020; 13: 1577–1590. 31607665 10.1016/j.jcmg.2019.09.006 PMC7148179

